# Parenthood and lower risk of suicide in women and men: the total Swedish population followed across adulthood

**DOI:** 10.1007/s00127-022-02321-y

**Published:** 2022-07-15

**Authors:** Alma Sörberg Wallin, Hugo Sjöqvist, Marina Dehara, Michael B. Wells, Jennifer Dykxhoorn, Kyriaki Kosidou, Christina Dalman

**Affiliations:** 1 Department of Global Public Health, Karolinska Institutet, Solnavägen 1C, 113 65 Stockholm, Sweden; 2 Clinical Epidemiology Division, Department of Medicine Solna, Karolinska Institutet, Stockholm, Sweden; 3 Department of Women’s and Children’s Health, Karolinska Institutet, Stockholm, Sweden; 4 Division of Psychiatry and Department of Primary Care and Population Health, UCL, London, UK; 5 Centre for Epidemiology and Community Medicine, Region Stockholm, Stockholm, Sweden

**Keywords:** Suicide, Parents, Life course, Cohort, Epidemiology

## Abstract

**Background:**

Previous studies suggest a protective effect of parenthood on suicide, but little is known about how the association may change across the lifespan, or in relation to sex, marital status or occurrence of psychiatric disorders.

**Methods:**

We followed a cohort of over 5 million Swedish women and men, from 1991 to 2011, up to max. age 75, for death by suicide using national registers. Information on childbirths/adoptions, potential confounders and modifying factors were obtained from national registers. We assessed the associations between parenthood and suicide across adulthood using within time-stratified Cox regression models, with parenthood as a time-dependent exposure.

**Results:**

Parents had a lower risk of suicide than non-parents across the lifespan, after adjusting for sociodemographic factors. The association was most pronounced in young adults, especially young women, but attenuated with increasing age and converged between sexes in older age groups. The lower risk of suicide over the life course was similar whether parents were married, unmarried or divorced, apart from married men; among them, parents only had a lower risk above age 55. The lower risk in parents was also evident in people with a history of psychiatric hospitalizations, but disappeared from age 55 in this population.

**Conclusion:**

The lower risk of suicide was present in both parents, was most pronounced in young adulthood and weakened with increasing age. Our results are consistent with a plausible mechanism where feelings of responsibility and connectedness are protective against suicide in parents.

**Supplementary Information:**

The online version contains supplementary material available at 10.1007/s00127-022-02321-y.

## Introduction

Suicide is a public health challenge worldwide, with around 800000 deaths reported annually [1]. While globally, more men die by suicide than women, with a ratio of three to one in high-income countries [1], women have higher rates of suicide attempt, self-harm [2, 3] and psychiatric disorders, such as depression [4].

One suggested explanation for the lower rates of suicide deaths in women is the role of motherhood [5]. Several studies on suicide rates in mothers and women without children support this notion [6–9], but studies on both mothers and fathers are scarce and have shown mixed results [10, 11]. However, our recent Swedish study consisting of 1.5 million adults showed a strong association between parenthood and low suicide risk in both women and men aged 25 to 44, even after controlling for a range of potential confounders [11].

However, the parental role changes across adulthood, especially as children become adults and therefore less reliant on their parents for support. Here, we therefore expand on our previous findings and examine the association of parenthood with suicide in both mothers and fathers over the life course.

The association between parenthood and lower suicide risk in men might be confounded by marital status, as studies on living arrangements suggest that men with a spouse do not gain any additional protection from having children in the household [12, 13], but research on this is sparse. Further, people with severe psychiatric disorders may have a different patterning of suicide risk factors than the general population [14], but no previous study has examined whether parenthood is protective against suicide among psychiatric patients.

In the present study, we followed the total Swedish population born 1936 to 1989 for 21 years, from 1991 to 2011, to investigate the associations between both father- and motherhood and the risk of suicide over the life course. We examined whether this association varies by marital status or diagnosis of a psychiatric disorder.

## Methods

### Study population

Data were retrieved from Psychiatry Sweden, a database linkage of national and local registers in Sweden, via the unique personal identification number held by every Swedish resident. The present study is based on the 6,004,335 people born from 1936 to 1989 who were alive and a Swedish resident by the end of 1990, regardless of parental status by the start or end of the study period. After exclusions (Supplementary Figure S1), 5,824,198 people (97%) were kept for analysis. They were followed in registers from the beginning of 1991, or the year they turned 20, up to the end of 2011, emigration or death due to any cause, whichever came first. By using a time-varying design, data on parenthood, suicide death and exit from this study were updated every year.

To investigate the distribution of suicides by the age of the child (post hoc, motivated by the different patterns in the main results for women and men over the life course), we looked at an additional population of all parents who died by suicide from 1961 onwards (quality data on causes of death starts from this year), and before their youngest child turned 30 years, to ensure that they could be followed for an equal length of time. We included only those parents (*n* = 11,858) who had their youngest child at least 30 years before the end of the study period in 2011. Their birth years ranged from 1936 to 1962.

### Ethical considerations

Ethical approval was granted by the Regional Ethical Board in Stockholm (decision reference numbers 2010/1185-31/5 and 2013/1118-32). Due to the study design, informed consent from the participants was not required.

### Data

#### Exposure: parenthood

Parenthood was defined by having at least one child (biological or adopted) identified as the participant’s offspring in the Multi-Generation Register, which has data available for nearly 100% of mothers and 98% of fathers for children born after 1961 [15] until 2011 in the Psychiatry Sweden database. Parental status (parent or non-parent) was defined at the baseline, with a yearly update thereafter until the end of 2011.

### Outcome: suicide

Suicide was defined from the underlying cause of death in the Cause of Death Register [16] and classified according to the international classification of disease (ICD) through the following diagnoses: intentional suicide and self-harm (E950-E959 [ICD-9, 1991–1996] and X60–X84 [ICD-10, 1997–2011]) and undetermined intent (E980-E989 [ICD-9] and Y10–Y34 [ICD-10]). Events of undetermined intent were also included, which is standard in register-based studies to minimize misclassification bias, since these events are likely to be suicides [17, 18].

### Covariates

The covariates were chosen by their documented association with suicide risk [5, 10, 19]. Information on sex and country of birth at baseline, and marital status with yearly updates, was retrieved from the Total Population Register [20]; educational attainment at baseline from the Longitudinal Integration Database for Health Insurance and Labor Market Studies [21] and any psychiatric diagnosis between 1973 (when these data became of sufficient quality) and end of the study period in 2011 from the National Inpatient Register [22]. We acquired deprivation index, population density and proportion of migrants/children of migrant(s) in the participants’ residential area in the year before baseline using the Small Area Marketing Statistics (SAMS) areas, maintained by Statistics Sweden in the Total Population Register [23]. Details are provided in Supplementary Table S1.

### Statistical analyses

We investigated the association between parenthood and death by suicide between 1991 and 2011 using a survival analysis approach with Cox regressions to calculate hazard ratios (HR) with 95% confidence intervals (CI), with parenthood as a time-varying variable with yearly updates. Parenthood was dichotomized into parent and non-parent. We used age as the underlying time scale and additionally adjusted for participants’ birth year in the basic model. Interaction with participants’ sex (parenthood*sex) was assessed and established (*p* < 0.001) and all analyses were subsequently stratified by sex. We repeated the analyses with additional adjustment for country of birth, educational level and neighbourhood characteristics (population density, deprivation and proportion of residents who were migrants/children of migrant(s)). Lastly, we repeated the analyses with stratification by psychiatric diagnosis and marital status for possible effect modification. The cohort was followed with yearly updates from entering this study in 1991, or at a minimum age of 20, to the year of emigration, death or to the end of the study in 2011, whichever came first. We performed within-time-stratified analyses with 5 year intervals to take the non-proportional effect over time into consideration, and in 15-year intervals in analyses stratified by marital status to retain statistical power. In sensitivity analyses, we (a) excluded deaths of undetermined intent from the outcome, (b) adjusted for competing risks by other causes of death [24] and (c) restricted the psychiatric population to those with an inpatient psychiatric diagnosis on the past five years only to check for information bias, because older age groups had no such data available from younger years. The data management and descriptive calculations were performed in SAS v. 9.4, the regression analyses were performed in Stata v. 15.1 and Fig. 2 was generated in R v. 3.5.1. using the ggplot2 package [25].

## Results

Between 1991 and 2011, there were 21,861 suicides (375 per 100,000) in the study population; 6,241 in women and 15,620 in men. The characteristics of the participants differed between those who died by suicide and those who did not (Table 1). People who died by suicide were more likely than others to have only basic education, have a psychiatric diagnosis from inpatient care and to be unmarried or divorced/widowed at the end of the study.

**Table 1:** Characteristics of the study sample and their distribution by suicide or no suicide during the study period

Women, *N* = 2,847,471	Total		No suicide*		Suicide*	
	N/mean	%/(SD)	N/mean	%/(SD)	N/mean	%/(SD)
Birth year, mean	1962	(15)	1962	(15)	1956	(13)
Country of birth, other than Sweden						
Nordic country	105,307	4	104,851	4	456	7
Other country	150,771	5	150,396	5	375	6
Population density in residential area**						
Low, rural (< 20th percentile)	541,281	19	540,378	19	903	14
Medium–low (20–40th percentile)	561,092	20	560,041	20	1051	17
Medium (40–60th percentile)	572,938	20	571,877	20	1061	17
Medium–high (60–80th percentile)	582,925	20	581,542	20	1383	22
High, urban (> 80th percentile)	589,235	21	587,392	21	1843	30
Proportion migrants/children of migrants in residential area**						
Low, mostly Swedish origin (< 20th percentile)	539,742	19	538,875	19	867	14
Medium–low (20–40th percentile)	561,953	20	560,894	20	1059	17
Medium (40–60th percentile)	575,666	20	574,481	20	1185	19
Medium–high (60–80th percentile)	580,799	20	579,435	20	1364	22
High, mostly non-Swedish origin (> 80th percentile)	589,311	21	587,545	21	1766	28
Deprivation in residential area**						
Low, non-deprived (< 20th percentile)	522,476	18	521,651	18	825	13
Medium–low (20–40th percentile)	580,721	20	579,634	20	1,087	17
Medium (40–60th percentile)	616,354	22	614,994	22	1360	22
Medium–high (60–80th percentile)	569,366	20	567,991	20	1375	22
High, deprived (> 80th percentile)	558,554	20	556,960	20	1594	26
Educational level**						
Primary school < 9 years	205,832	7	205,146	7	686	11
Primary school, 9 years	461,408	16	459,927	16	1481	24
Secondary school, 2–4 years	1,705,404	60	1,702,378	60	3026	48
Post-secondary education, 2 years or more	474,827	17	473,779	17	1048	17
Psychiatric disorder (ever)	300,073	11	295,782	10	4291	69
Marital status (at end of study)						
Unmarried	1,264,864	44	1,261,860	44	3004	48
Married	1,064,089	37	1,062,879	37	1210	19
Divorced/widowed	518,518	18	516,491	18	2027	32

Men, *N* = 2,976,727	Total		No suicide*		Suicide*	
	N/mean	%/(SD)	N/mean	%/(SD)	N/mean	%/(SD)
Birth year, mean	1962	(15)	1962	(15)	1957	(13)
Country of birth, other than Sweden						
Nordic country	89,335	3	88,495	3	840	5
Other country	169,263	6	168,496	6	767	5
Population density in residential area**						
Low, rural (< 20th percentile)	622,621	21	619,245	21	3376	22
Medium–low (20–40th percentile)	604,494	20	601,701	20	2793	18
Medium (40–60th percentile)	592,843	20	590,228	20	2615	17
Medium–high (60–80th percentile)	583,354	20	580,334	20	3020	19
High, urban (> 80th percentile)	573,415	19	569,599	19	3816	24
Proportion migrants/children of migrants in residential area**						
Low, mostly Swedish origin (< 20th percentile)	600,169	20	597,190	20	2979	19
Medium–low (20–40th percentile)	596,650	20	593,944	20	2706	17
Medium (40–60th percentile)	596,304	20	593,457	20	2847	18
Medium–high (60–80th percentile)	587,298	20	584,240	20	3058	20
High, mostly non-Swedish origin (> 80th percentile)	596,306	20	592,276	20	4030	26
Deprivation in residential area**						
Low, non-deprived (< 20th percentile)	548,199	18	546,341	18	1858	12
Medium–low (20–40th percentile)	609,345	20	606,686	20	2659	17
Medium (40–60th percentile)	647,241	22	643,859	22	3382	22
Medium–high (60–80th percentile)	595,983	20	592,399	20	3584	23
High, deprived (> 80th percentile)	575,959	19	571,822	19	4137	26
Educational level**						
Primary school < 9 years	261,070	9	258,955	9	2115	14
Primary school, 9 years	533,967	18	530,075	18	3892	25
Secondary school, 2–4 years	1,722,999	58	1,715,257	58	7742	50
Post-secondary education, 2 years or more	458,691	15	456,820	15	1871	12
Psychiatric disorder (ever)	336,139	11	328,004	11	8135	52
Marital status (at end of study)***						
Unmarried	1,492,493	50	1,483,123	50	9370	60
Married	1,095,771	37	1,092,885	37	2886	18
Divorced/widowed	388,463	13	385,099	13	3364	22

^*^All *p*-values from Chi-square and *t*-tests for group differences are < 0.001

^**^Measured at baseline, at age min. 19 years

^***^Marital status is modelled as time varying with yearly updates in the analyses; the single-time measure was used for this table only

**Fig. 1:**
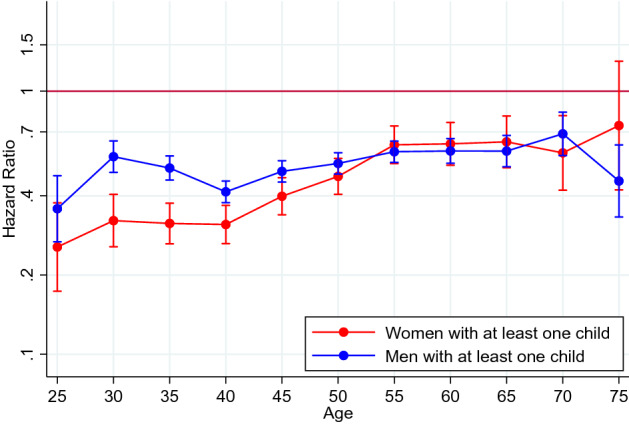
Association between parenthood and suicide 1991–2011 in women and men, by age group (the age denotes the highest age reached in each 5-year interval). Hazard ratios (HRs) with 95% confidence intervals associated with having at least one child, compared with having no children (at HR = 1). Adjusted for birth year, country of birth, attained education and neighbourhood characteristics (deprivation, population density and proportion of migrants/children of migrants)

In models analysing the risk of suicide in 5-year intervals across adulthood, adjusting for the potential confounders, the risk of suicide was lower in parents with children in all age groups, compared to people without children (although the confidence interval included 1 for women in the oldest age group, 70–75; Fig. 1). However, the pattern across age groups differed by sex. Mothers had particularly low risks in early adulthood, but the risk reduction attenuated with increasing age up to age 55, and then stabilized. For example, at age 20 to 25, mothers had about a 76% lower risk of suicide (adjusted HR [aHR] 0.24, 95% CI 0.16–0.36) than women with no children, while at age 50 to 55, the corresponding risk reduction was 39% for mothers (aHR 0.61, 95% CI 0.51–0.71). On the other hand, the lower risk in fathers was more stable over the life course, with 67 to 51% lower risks compared with men with no children over the same period. The exact estimates for the fully adjusted models, and the models adjusted for birth year only, are shown in Supplementary Table S2.

**Fig. 2:**
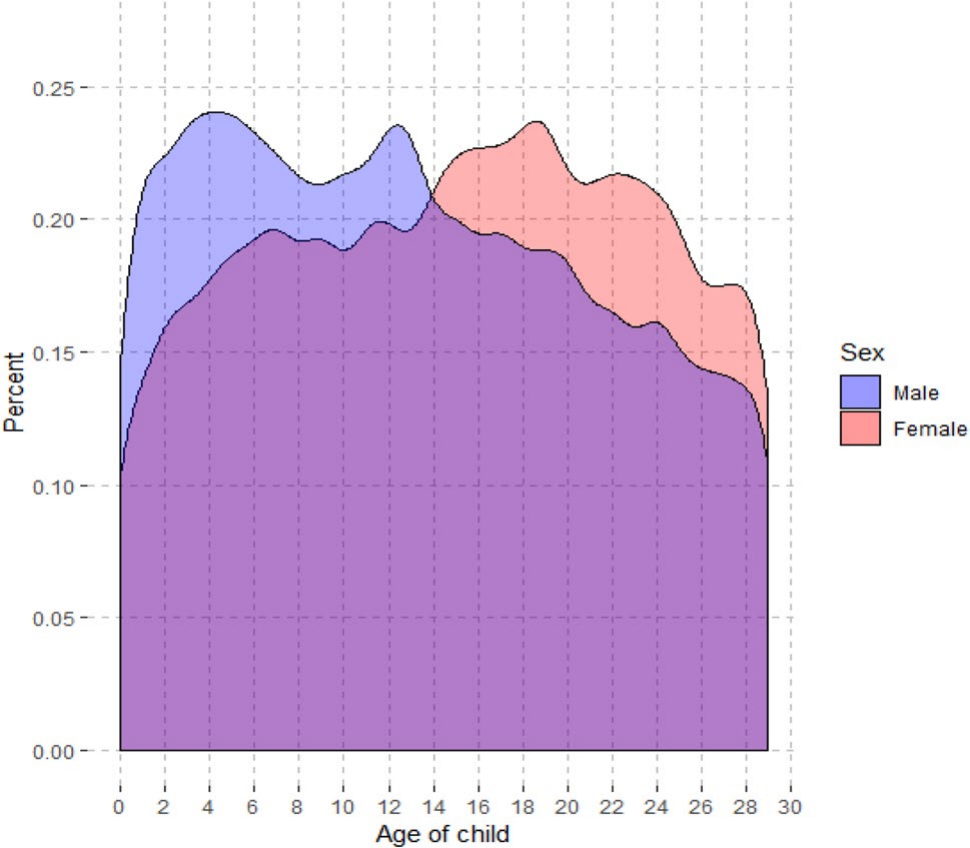
Suicide in parents, distributed by the age of their youngest child, in women and men who died by suicide before the child turned 30 years old

As shown in Fig. 2 (note: this population is not identical with the population in the main analysis, as detailed in Methods), the number of suicides in mothers was relatively low during infancy, preschool and elementary school ages but increased during adolescence. By contrast, suicide in fathers peaked during the preschool years and at the beginning of the teenage years.

**Fig. 3:**
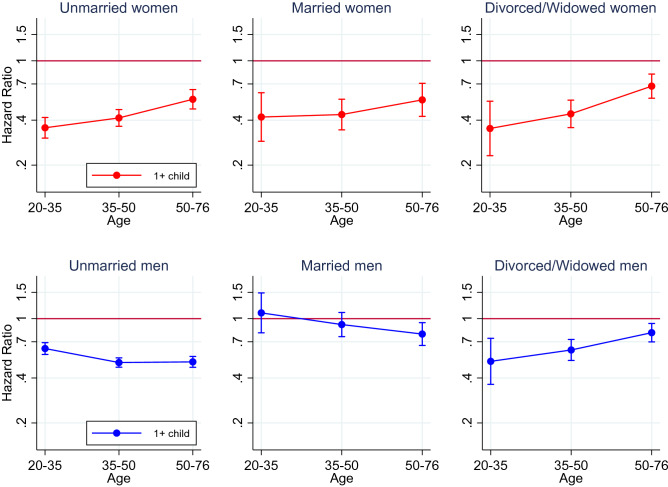
Association between parenthood and suicide, stratified by marital status. HRs with 95% CIs associated with having at least one child, compared with having no children. Adjusted for birth year, country of birth, attained education and neighbourhood characteristics (deprivation, population density and proportion of migrants/children of migrants)

When stratified by marital status, the association between parenthood and suicide followed roughly the same pattern for the unmarried, married and divorced/widowed women (Fig. 3). However, in married men in age groups 20 to 35 and 35 to 50, there was no association between parenthood and suicide; only in the age group 50 to 75 parenthood did confer a slightly lower risk. By contrast, parenthood was associated with a substantially lower risk of suicide in all age groups in unmarried men, and in divorced/widowed men, the association was strongest in the youngest age group and attenuated towards the oldest age group.

**Fig. 4:**
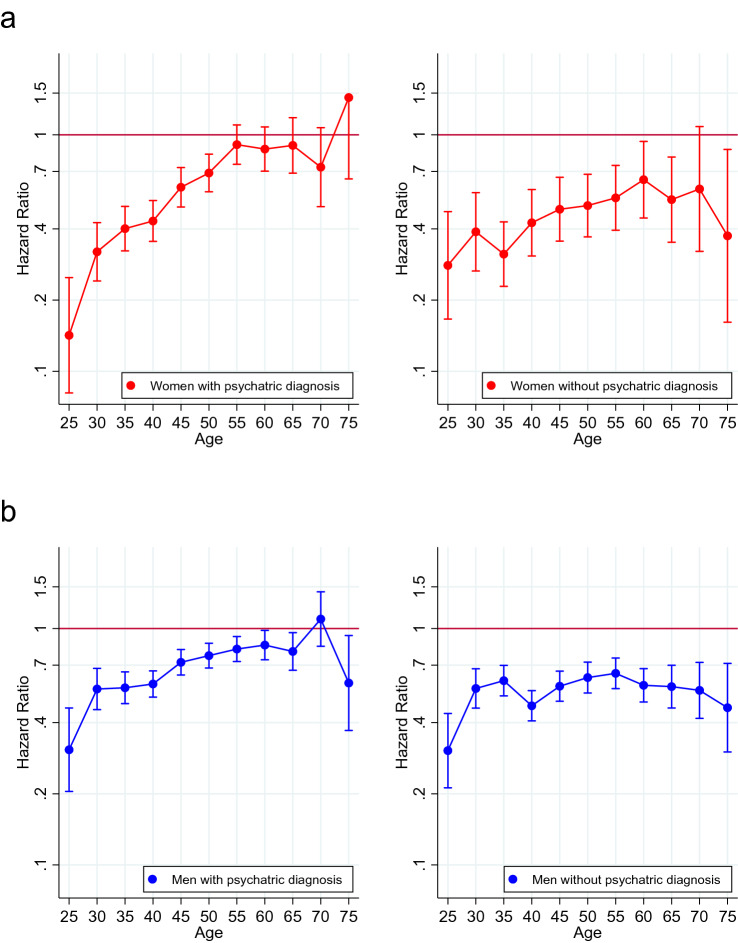
Association between parenthood and suicide 1991–2011, by age group, stratified by lifetime psychiatric diagnosis in inpatient care, in **a** women and **b** men. HRs with 95% CIs associated with having at least one child, compared with having no children. Adjusted for birth year, country of birth, attained education and neighbourhood characteristics (deprivation, population density and proportion of migrants/children of migrants)

When stratified by psychiatric diagnosis in inpatient care, the pattern over the lifespan was generally stable in both women and men without a psychiatric diagnosis (Fig. 4). By contrast, in women with a psychiatric diagnosis, the difference in suicide risk between mothers and non-mothers diminished markedly with increased age. In women with a psychiatric diagnosis, at age 50 and older, mothers did not have a distinctly lower risk than non-mothers. In men with psychiatric diagnoses, too, the lower risk in fathers seemed to lessen with age.

### Sensitivity analyses

After excluding cases of undetermined intent from the suicide outcome, the estimates and overall trends were similar to the results from the main analysis (Supplementary Table S3). Adjustment for competing risk of death also produced similar results (Supplementary Table S4). Restricting the psychiatric population to people with a psychiatric diagnosis in the past five years yielded similar but slightly weaker associations than using inpatient data from all available years (Supplementary Figure S2).

## Discussion

In this large, longitudinal study of the Swedish population, we found that parenthood was strongly associated with low suicide risk, but the association attenuated with age. Moreover, the life course patterns differed by sex. In women, the association between parenthood and low suicide risk was strong in young adulthood but attenuated after middle age, while in men, the association was more moderate but stable over the life course. The youngest child’s age seemed important for the age patterns. In mothers who died by suicide, death was most prevalent when the child had reached late adolescence, but in fathers, death was most prevalent in the child’s preschool and early teenage years. Marital status modified the association only among men, such that the association between parenthood and lower suicide risk was not present in young and middle-aged married men. In both women and men with a psychiatric diagnosis from inpatient care, the association diminished with increasing age and was not present from age 55 in women.

### Comparison with previous studies

The lower risk of suicide in parents compared with non-parents is in line with previous studies [6–10, 12, 26], including our recent study [11], but few included both women and men [10, 12]. To our knowledge, no previous studies have investigated the association between parenthood and suicide risk in women and men over the life course. However, one Norwegian study investigated the association between parental status and suicide in married women in different age groups [6]. They found no association between motherhood and risk of suicide in the youngest age group, 25–34 years, while in older age groups mothers had a lower risk than women without children. These results differ from our findings where motherhood was strongly associated with low suicide risk also in the younger age groups. Different methods, such as using parental status at baseline instead of allowing parental status to change over the follow-up as in the present study, might explain some of the contrasting results as many of the younger women may have become parents during the follow-up.

While the current findings of suicide rates of mothers and fathers are in line with a Danish study regarding the child’s age, our findings based on marital status differ [10]. In that study, a lower risk with an increasing number of children was evident in married and cohabiting men, even after adjustment for other risk factors. However, in single men, parenthood was not associated with a lower risk, and single men having two to four children even had a slightly increased risk compared to their childless counterparts. We are not aware of any previous studies on the association between parenthood and suicide in psychiatric patients over time.

### Possible explanations

Parents with suicidal thoughts and behaviours might view their responsibility for their children as a reason to stay alive [27], and in a Swedish study of an inventory of reasons for living, women reported their concern for children as a greater reason to go on living than men did [28]. The Interpersonal Theory of Suicide asserts that a sense of belonging and social connectedness is protective against suicide [29]. Having and rearing children may indeed raise these feelings in both parents, relative to those without children, thus reducing their risk of suicide. Today, across the world and in Sweden, fathers take on more childrearing responsibilities than ever before, and therefore, future follow-ups will benefit from assessing if this further decreases fathers’ suicide risks, especially when their children are preschool age.

Parents’ health behaviours might also lend some protective effect. For example, findings in a recent study suggest that motherhood lowers the risk of alcohol use disorders [30]. Another possible explanation for a weaker association in men than in women is that socioeconomic factors, including unemployment and financial problems, seem more important for suicide risk for men than for women [3, 19]. Thus, even if parenthood has a protective effect in fathers, other conditions might outweigh this effect.

Marriage and partnership have been reported as stronger protective factors against suicide in men than in women [8, 10, 12, 13, 31]. Thus, even if parenthood is important for men’s social connectedness, parenthood might not add much protection over and above the marital relationship. By contrast, for single fathers, child connectedness might be a more important source of connectedness. A limitation to the current study is that those parents who cohabit are categorized within the unmarried group, as no available item would let us separate these groups. However, parenthood was almost as strongly associated with lower suicide risk in divorced and widowed men, and having children is unlikely to proxy a relationship in divorced and widowed people. Thus, we may speculate that parenthood is indeed more protective in single than in married men. In line with the Swedish policies about gender-equal parenting, Swedish fathers are active parents even if they are not living with the co-parent [32], and in a previous large-scale study, single fathers had a higher risk of suicide than cohabiting fathers only if they did not have custody [26].

In the population with a psychiatric diagnosis from inpatient care, the association between parenthood and suicide was weakened for all with increasing age, but this attenuation was more pronounced among women. A possible explanation is that, as previously mentioned, concern for children might be a strong reason for people with suicidal thoughts and behaviours to refrain from suicide [27]. When the child approaches independence, this concern might weaken. The lack of an association with parenthood in older age groups might indicate a need for other ways to enhance a sense of connectedness and social responsibility in mentally ill people who have lost their family ties due to older age. The particularly low risk in young mothers among people with a psychiatric diagnosis might also be due to the close professional monitoring in the postpartum period and later, which is traditionally more focused on mothers than fathers [33]. Mortality displacement (a “harvesting effect”) could also attenuate the association over time. However, suicide is relatively common in older age groups and other risk factors may be important then than in younger years [34].

Finally, while the observed associations may be due to a causal effect of parenthood on reducing suicide risk, the associations may also be due to selection into parenthood. The slight attenuation from adjusting for confounding factors suggests a limited effect of selection. In our recent study on a subsample of the population in the present study, following them from age 25 to 45, we could adjust for a greater range of factors, including socioeconomic and labour market factors, and individual characteristics, such as personality aspects and cognitive ability in men. These adjustments also had a quite limited effect on the associations [11]. Moreover, the changes over the life course in women in the present study suggest an actual protective effect, at least in young women. However, the mechanisms underlying the associations, and possible residual selection effects, need to be investigated in future studies. More insight into the mechanisms may also inform on how people without children can find protective mechanisms in other ways.

### Strengths and limitations

The present study was based on the total Swedish population with birth years 1936 to 1989, followed for suicide in national records for a time period of 21 years, which enabled us to compare women and men at different ages over the life course. Register linkage provided reliable data on birth and adoptions of children, deaths registered as suicide and demographic factors for control for selection and effect modification. The time-varying design with yearly updates of parental status and suicide was particularly important since suicide is common in the fertile years of people’s lives, and parental status changes in most participants over the study period.

However, the use of registers limits the information available. We had no data on other indicators of selection into parenthood, such as relationship difficulties, or on other modifying factors, such as parenting behaviours and parenting opportunities. Although parenting in Sweden is relatively gender equal, women are often reported to take more responsibility in childrearing [35]. Likewise, we had no data to investigate potential mechanisms for a protective effect of parenthood on suicide, such as social connectedness, better health behaviours, or connectedness and sense of responsibility for the children, all of which might lower suicide risk [27, 29, 36]. The register data are limited to information on legal sex as opposed to e.g. gender. People with a gender identity different from their legal sex, who may comprise 0.5% of adults [37], have a higher suicide rate than the general population [37] and generalization to them is severely compromised. Given the policies of gender equality in parenting in Sweden and other Scandinavian countries, generalizations to other countries must be done with caution. When stratifying by psychiatric diagnosis in inpatient care, some individuals may have been misclassified as not having a psychiatric disorder because they died by suicide before receiving a diagnosis or were treated only primary or outpatient care, which might have artificially inflated or deflated the risk in the group without psychiatric disorders. Moreover, the association between parenthood and suicide may differ by the type of psychiatric disorder [38], but we did not have the statistical power to investigate differences between subgroups of psychiatric disorders over the life course. Lastly, we only had information on psychiatric diagnosis in inpatient care and could not investigate any possible effect of perinatal or postpartum depression which is most often treated in outpatient or primary care. It is possible that the risk of suicide is even lower in parents free from these conditions. Nevertheless, suicide in the first year of parenthood was very rare and it is unlikely that these conditions had any major effects on the main results.

### Conclusions

Parenthood was associated with a reduced suicide risk throughout adulthood, but the association weakened with increasing age. This attenuation was especially apparent in women; in young adulthood the low suicide risk was more marked in women then in men, but in older age groups there were no substantial sex differences. In people with psychiatric hospitalizations the association weakened substantially across adulthood, with only a small difference between parents and non-parents remaining in older age groups. Although more research on the mechanisms of the association is needed, the results are compatible with the traditional suggestions that parenthood is protective against suicide through the sense of connectedness and responsibility that parenthood may entail.

## Supplementary Information

Below is the link to the electronic supplementary material.

Supplementary file1 (DOCX 73 KB)

## Data Availability

Data cannot be shared publicly under the terms and conditions of ethical approval for Psychiatry Sweden. This research had ethical approval obtained by the Stockholm Regional Ethical Review Board (decision reference numbers 2010/1185‐31/5 and 2013/1118‐32). Please contact the Stockholm Regional Ethical Review Board (https://etikprovningsmyndigheten.se/) for information about access to Swedish register data for researchers who meet the criteria for access to confidential data. The code used for data preparation and analyses is available at https://osf.io/2ye53/.
