# Supplementary material for: Parenthood and lower risk of suicide in women and men: the total Swedish population followed across adulthood
Source: Soc Psychiatry Psychiatr Epidemiol. 2022 Jul 15;57(11):2241–50. doi: 10.1007/s00127-022-02321-y (PMC9636107; doi:10.1007/s00127-022-02321-y)
Supplement: Supplementary file 1 — Supplementary file1 (DOCX 73 KB) [file 127_2022_2321_MOESM1_ESM.docx]

Supplementary material.


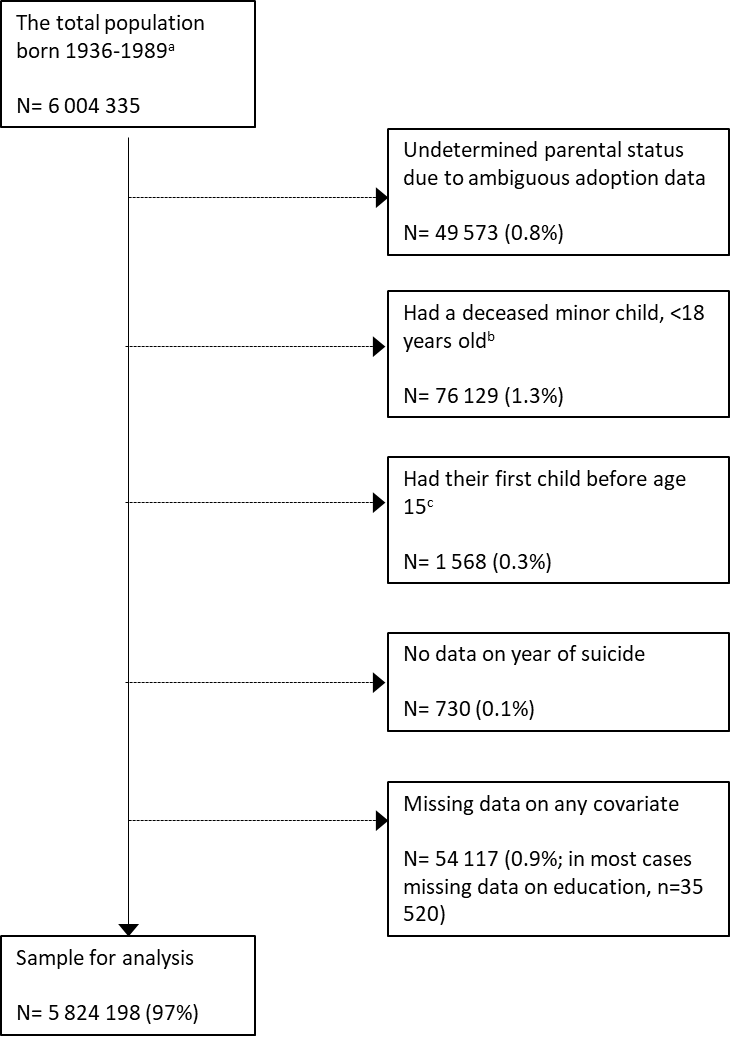


Figure S1. Flow chart of study sample. a) Alive and living in Sweden 1990; b) These participants were excluded due to the radical change in the parental role and the acute risk of suicide associated with the loss (Qin & Mortensen, 2003) ; c) Some of these were registered as becoming parents at a very young age (<10 years) and probably misclassified.

Table S1. Additional information on covariates

| **Variable** | **Type** | **Source** | **Comment** |
| --- | --- | --- | --- |
| Residential area/ neighbourhood variables:  - deprivation index  - proportion migrants/ children of migrants  - population density | Categorical: Quintiles. | Statistics Sweden’s “Small Area Marketing Statistics” (SAMS) classification system, and the Total Population Register (TPR) for information on country of origin. | Retrieved the year before study entrance. The “Small Areas for Market Statistics” is a classification of demographically homogeneous geographical areas into 9,208 neighbourhoods. The participants were grouped into quintiles by deprivation index, proportion of migrants and/or children of at least one migrated parent (by linkage to population data by region of origin in the TPR), and population density (calculated by dividing the number of residents by the geographical land area) in their neigbourhood. Participants with no registered residence (n= 13 940; 0.2%) were placed in a separate category. |
| Education | Categorical: Primary school <9 years; Primary school of 9 years; Secondary school; Post-secondary education. | The Longitudinal Integration Database for Health Insurance and Labor Market Studies (data available from 1990 onwards). | Retrieved the year before study entrance. |
| Psychiatric diagnoses | Binary: any; none (between 1973-2011). | The National Inpatient Register (data on psychiatric inpatient care since 1973). | ICD codes 295-319 (ICD-9, 1990-1996) and F00–F99 (ICD-10, 1997 onwards). Psychiatric diagnosis was retrieved from all years available until the end of the study and dichotomized into any or none, rather than updated yearly. This was because severe psychiatric disorders are not limited to the timing of hospitalization but may be viewed as long-term conditions that are often managed outside of inpatient care. |
| Marital status | Categorical: Unmarried; Married; Divorced/widowed. | The TPR. | Retrieved for every year of follow-up and modelled as a time-varying variable in the stratified Cox model. No information on cohabitation was available. Divorced/widowed was combined due to a limited number of widowed people. |

Table S2. Associations between parenthood and suicide 1991-2011, in models with stepwise adjustments.

|  | Women |  | Men |  |
| --- | --- | --- | --- | --- |
|  | Model 1* | Model 2** | Model 1* | Model 2** |
| Age | HR (95% CI) | HR (95% CI) | HR (95% CI) | HR (95% CI) |
| 20-25 | 0.33 (0.23; 0.49) | 0.24 (0.16; 0.36) | 0.37 (0.28; 0.50) | 0.33 (0.25; 0.44) |
| 25-30 | 0.40 (0.32; 0.51) | 0.35 (0.28; 0.44) | 0.61 (0.53; 0.70) | 0.56 (0.49; 0.64) |
| 30-35 | 0.32 (0.27; 0.38) | 0.31 (0.26; 0.37) | 0.51 (0.45; 0.56) | 0.51 (0.46; 0.57) |
| 35-40 | 0.30 (0.25; 0.35) | 0.31 (0.26; 0.37) | 0.38 (0.35; 0.42) | 0.41 (0.37; 0.45) |
| 40-45 | 0.38 (0.32; 0.45) | 0.40 (0.34; 0.47) | 0.45 (0.41; 0.49) | 0.49 (0.45; 0.54) |
| 45-50 | 0.42 (0.36; 0.49) | 0.47 (0.40; 0.55) | 0.47 (0.43; 0.52) | 0.54 (0.49; 0.59) |
| 50-55 | 0.54 (0.46; 0.63) | 0.61 (0.51; 0.71) | 0.52 (0.47; 0.57) | 0.59 (0.53; 0.64) |
| 55-60 | 0.54 (0.45; 0.64) | 0.64 (0.53; 0.77) | 0.52 (0.47; 0.58) | 0.59 (0.53; 0.66) |
| 60-65 | 0.54 (0.43; 0.68) | 0.63 (0.50; 0.78) | 0.50 (0.44; 0.57) | 0.58 (0.50; 0.66) |
| 65-70 | 0.52 (0.38; 0.72) | 0.58 (0.42; 0.80) | 0.59 (0.49; 0.71) | 0.68 (0.56; 0.82) |
| 70-75 | 0.60 (0.35; 1.05) | 0.74 (0.42; 1.30) | 0.42 (0.31; 0.57) | 0.47 (0.34; 0.64) |

* Adjusted for birth year.

** Main analysis: adjusted for birth year, attained education, country of birth, and neighbourhood characteristics (deprivation, population density, and proportion of foreign-born residents).

Table S3. Sensitivity analysis. Association between parenthood and suicide; not including cases with undetermined intent.

|  | Women | Men |
| --- | --- | --- |
| Age | HR (95% CI)* | HR (95% CI)* |
| 20-25 | 0.22 (0.14; 0.34) | 0.34 (0.25; 0.47) |
| 25-30 | 0.35 (0.27; 0.46) | 0.53 (0.45; 0.62) |
| 30-35 | 0.28 (0.23; 0.35) | 0.54 (0.48; 0.61) |
| 35-40 | 0.29 (0.24; 0.35) | 0.44 (0.39; 0.49) |
| 40-45 | 0.40 (0.33; 0.48) | 0.55 (0.50; 0.62) |
| 45-50 | 0.45 (0.38; 0.54) | 0.59 (0.53; 0.66) |
| 50-55 | 0.59 (0.49; 0.71) | 0.60 (0.54; 0.67) |
| 55-60 | 0.61 (0.49; 0.76) | 0.60 (0.53; 0.68) |
| 60-65 | 0.72 (0.55; 0.96) | 0.56 (0.48; 0.65) |
| 65-70 | 0.58 (0.40; 0.84) | 0.72 (0.58; 0.89) |
| 70-75 | 0.83 (0.41; 1.71) | 0.40 (0.29; 0.56) |

* Adjusted for birth year, attained education, country of birth, and neighbourhood characteristics (deprivation, population density, and proportion of foreign-born residents).

Table S4. Sensitivity analysis. Association between parenthood and suicide, adjusting for competing risks.

|  | Women | Men |
| --- | --- | --- |
| Age | HR (95% CI)* | HR (95% CI)* |
| 20-25 | 0.24 (0.16; 0.37) | 0.33 (0.24; 0.45) |
| 25-30 | 0.35 (0.27; 0.44) | 0.56 (0.48; 0.65) |
| 30-35 | 0.31 (0.26; 0.37) | 0.51 (0.46; 0.57) |
| 35-40 | 0.31 (0.26; 0.37) | 0.41 (0.37; 0.45) |
| 40-45 | 0.40 (0.34; 0.48) | 0.49 (0.45; 0.54) |
| 45-50 | 0.47 (0.40; 0.55) | 0.54 (0.49; 0.59) |
| 50-55 | 0.61 (0.52; 0.72) | 0.59 (0.54; 0.65) |
| 55-60 | 0.64 (0.53; 0.77) | 0.59 (0.53; 0.66) |
| 60-65 | 0.63 (0.50; 0.79) | 0.58 (0.51; 0.67) |
| 65-70 | 0.58 (0.42; 0.81) | 0.69 (0.57; 0.83) |
| 70-75 | 0.74 (0.43; 1.29) | 0.47 (0.34; 0.64) |

* Adjusted for birth year, attained education, country of birth, and neighbourhood characteristics (deprivation, population density, and proportion of foreign-born residents).

Figure S2. Sensitivity analysis. Association between parenthood and suicide 1991-2011 in a) women and b) men, by age group, stratified by psychiatric diagnosis in the past five years.
